# A study of the effects of thematic language teaching on the promotion of multimedia design students’ listening and speaking skills

**DOI:** 10.3389/fpsyg.2022.915145

**Published:** 2022-09-16

**Authors:** Sheng-Kai Yin

**Affiliations:** Department of Digital Multimedia Design, Cheng Shiu University, Kaohsiung, Taiwan

**Keywords:** thematic language teaching, listening, speaking skill, learning anxiety, traditional teaching model

## Abstract

Previously, language teaching has been focused on the passive learning of the alphabet. In addition, the research on teaching listening and speaking skills was limited. Listening skill is the key to learning a language, and speaking is the first explicit behavior of language. In order to improve language skills which are emphasized in new curriculum guidelines, student-centered thematic language teaching is considered as valuable. Through this, the concepts of multiple intelligences and curriculum integration were re-emphasized. An experimental design was adopted in the current study. This study was conducted with the participation of 224 students from the Department of Multimedia Design in universities in the south of Taiwan. The research data were collected between September 2021 and December 2021. The implementation process took 16 weeks (a total of 48 h) of thematic language teaching. The research results revealed 1. significant positive effects of thematic language teaching on listening, 2. significant positive effects of thematic language teaching on speaking skill, and 3. significant negative effects of thematic language teaching on learning anxiety. According to the results, it is expected that this study can help multimedia design students improve their listening and speaking skills as well as core language skills.

## Introduction

Under the information explosion in the global village with complicated and close connections of worldwide networks, English, as a *lingua franca*, becomes an important skill to enter the global world. Solgi and Tafazoli (2018) suggested that children learn a language in a natural context by first listening to the target language, paying conscious attention to the language, and then trying to express themselves in the target language after a period of contact. Listening skill is the first challenge in the process of language learning. Sälzer and Roczen (2018) indicated that language learning begins with communication experience. Adults stimulate children’s language acquisition device through different sounds, expressions, or movements. The children are constantly encouraged to speak and communicate. In this way, they learn new vocabulary. For this reason, listening and speaking play a crucial role at the beginning of language learning. Lin and Tseng (2020) pointed out that English classes used to focus on textbooks. They also stated that the emphasis on English teaching was more on vocabulary learning, while the training of English listening and speaking skills was not in the spotlight. The teaching content or materials were disconnected from real life, and students received little language stimulation. Therefore, without rich vocabulary and the lack of authentic situations, it became more difficult to use language to communicate. Blazar (2015) suggested listening and speaking skills as the keys to learning good English. To improve the effectiveness of English learning, listening and speaking problems should be overcome. English learning strategies are classified into direct strategies and indirect strategies. According to past scholars’ research and classification of language strategies, Oxford (1990) classified language learning strategies into direct and indirect strategies. The former referred to strategies directly related to learning itself and target language, including memory strategies, cognitive strategies, and compensation strategies. The latter referred to strategies to help and manage language learning, containing metacognitive strategies, affective strategies, and social strategies. Listening is the key to learning a language, and speaking is the first explicit behavior of language (Maulana et al., 2020). In order to improve language skills which are emphasized in new curriculum guidelines, student-centered thematic language teaching is considered as valuable. Through this, the concepts of multiple intelligences and curriculum integration were re-emphasized.

Thematic teaching has been hotly debated in education in recent years. This teaching method refers to a student-centered learning model, in which teachers and students discuss a learning topic that corresponds to students’ learning experiences or life backgrounds. During these discussions, they combine knowledge and skills from different areas, and explore and discuss the practical application. Teachers provide scaffolding through multiple instructional activity designs and teaching strategies, and they arrange flexible learning hours and learning content to meet the learning needs of students and achieve the best of learning effectiveness (Bezerra Fagundes, 2016). In this process, the interaction between teachers and students is emphasized to provide a comprehensive learning experience. Thematic teaching emphasizes awareness, communication and interaction, and teamwork. By doing so, it aims to achieve “spontaneity, interaction, and common good” (Haryanto et al., 2018). Thematic learning allows students to explore the real world, identify problems, and continue to search for solutions. The openness of students to participate in the curriculum design allows teachers to recognize the shortcomings and promote active learning. It is expected that through interaction with teachers, students’ thinking, communication, collaboration, and interdisciplinary skills will be improved to survive in the ever-changing world (Chen and Chang, 2018). Finland, leading the education trend, proposed Grade 1–9 new curriculum guidelines in 2016, stressing on phenomenon-based learning and allowing students exploring the real world, discovering problems, and further finding out solutions. Meanwhile, students were open to participate in the curriculum design for students knowing the insufficiency, enhancing active learning, as well as cultivating the thinking and communication, collaboration, and interdisciplinary ability through the interaction with teachers in order to face the constantly updated future world (Huang, 2018). Nevertheless, most previous research on thematic teaching discussed the overall planning of instruction and the preparation of activities (Romanova, 2017). In addition, the considerations in thematic teaching design were also at the forefront (Chumdari and Budiyono, 2018; Hennessey et al., 2018). This study can fill in a gap in the literature by providing concrete evidence. In this respect, with the expectation of helping multimedia design, students improve their listening and speaking skills and develop their core language skills.

## Literature review and hypothesis

Yeh and Lan (2018) stated that story-based thematic language teaching can improve students’ listening comprehension, learning attitude, and language learning outcomes. Story-based thematic language teaching can promote students’ interests and teacher-student interaction, improve students’ listening comprehension skills, reduce learning anxiety, and promote listening motivation. Students stated that song-based thematic language teaching enabled them to learn more vocabulary and helped them to memorize vocabulary effectively. Wardani et al. (2020a) mentioned that using video-based thematic language teaching can enhance students’ listening comprehension and learning attitude. When using video-based thematic language teaching, the content of the topic and the collaboration with peers should be properly adjusted according to the level of students. The students who were taught in the target language performed better in listening comprehension than the students who were taught with the traditional methods. Therefore, teaching in the target language was accepted and preferred by students. Normand and Burji (2020) pointed out the positive effect of thematic language teaching on learning attitude toward listening and on willingness to actively receive positive feedback. Thematic language teaching can reduce students’ learning anxiety, but does not help much with listening. During listening comprehension development, vocabulary input affects learners’ information processing in listening comprehension. Teaching in context can motivate learners to practice and promote their comprehension. Learners’ interests or life experiences were also taken into consideration when selecting the learning content for listening comprehension in order to promote the development of listening comprehension and positively influence learners’ motivation and interest in listening comprehension. Accordingly, the following hypothesis was proposed in this study.

*H1*: Phenomenon-based English teaching method presents significant effects on English listening.

*H1-0*: Phenomenon-based English teaching method shows remarkably negative effects on English listening.

*H1-1*: Phenomenon-based English teaching method reveal notably positive effects on English listening.

Lan and Liao (2018) considered that thematic language teaching can promote learners’ speaking and communication skills, learning attitude, and listening motivation. They added that it enables textbook publishers to design teaching materials that suit better to real-life communication situations. Sari et al. (2019) explained the factors that negatively affect students’ speaking acquisition as the focus on reading and writing, and lack of opportunities to use the target language. In the traditional language classrooms, most speaking activities are mechanical drills and lack meaningful communication and interaction. However, the use of thematic language teaching can improve students’ speaking skill, and meaningful teaching plays a primary role in the enhancement of students’ speaking skill. Ahn et al. (2019) stated that the use of thematic language teaching can promote G4 students’ speaking skills. In the task completion process, intensive use of language can enhance pupils’ speaking skills. The learning activities in thematic language teaching can improve students’ speaking performance, correctness, and emotional expression as well as promote students’ learning interests. Therefore, the following hypothesis was proposed in this study.

*H2*: Phenomenon-based English teaching method appears significant effects on English speaking ability.

*H2-0*: Phenomenon-based English teaching method presents remarkably negative effects on English speaking ability.

*H2-1*: Phenomenon-based English teaching method shows notably positive effects on English speaking ability.

Wu (2018) indicated that thematic language teaching can promote learners’ speaking and communication skills, learning attitude, and listening motivation. In addition, the publishers are forced to design teaching materials that are in line with the real communication situations and help reduce learners’ learning anxiety. Wardani et al. (2020b) stated that a thematic language instruction curriculum includes diverse teaching styles and methods to enrich students’ learning experience and improve students’ learning outcomes. Game elements can facilitate students’ learning in thematic language teaching. Through gaming, students with different learning achievement levels can reach significant effectiveness in thematic language teaching and their learning anxiety can be reduced. Moreno Martínez et al. (2018) mentioned that thematic learning can support cognitive, affective, subject-specific, and interdisciplinary learning, in addition to developing applications for life, and solving problems. Students had positive attitudes toward thematic learning and believed that the learning content is better organized and integrated to support learning. Students felt that the selection of themes was suitable for their level and interests which, in turn, improved their listening motivation and reduced their learning anxiety. As a result, the following hypothesis was proposed in this study.

*H3*: Phenomenon-based English teaching method reveals significant effects on learning anxiety.

*H3-0*: Phenomenon-based English teaching method appears remarkably positive effects on learning anxiety.

*H3-1*: Phenomenon-based English teaching method reveals notably negative effects on learning anxiety.

## Methodology

### Measurement of research variables

#### Listening skill

Following Hsu’s (2019) viewpoint that the focus of listening evaluation was on students’ vocabulary meaning, sentence, and simple dialog comprehension. The listening comprehension was evaluated through the selective listening, completing forms, paraphrasing sentences, delivering picture information, answering questions, role-playing, or gaming.

#### Speaking skill

Referring to the evaluation of learners’ speaking skill as proposed by Chang and Lin (2019), learners’ pronunciation was evaluated through imitation and repetition of vocabulary. The recitation of sentences can help the teacher to understand learners’ mastery of tone and rhythm as well as their fluency. Short answers and dialog can be used to evaluate learners’ context comprehension and completion of effective communication.

#### Learning anxiety

Following Lin and Tseng (2020), learning anxiety was defined as the negative emotions such as discomfort, nervousness, and worry in students’ language learning process that affect their learning performance. According to the study of Horwitz et al. (1986), learning anxiety is measured as a whole.

### Participants of the study

This study was conducted with the participation of 224 students from the Department of Multimedia Design in universities in the south of Taiwan. The implementation process took 16-week (a total of 48 h) of thematic language teaching. An experimental class was conducted through thematic language teaching, and the other class went on with the traditional teaching model for the one-semester experimental research. The retrieved questionnaire data were analyzed with SPSS. Analysis of variance was applied to test the hypotheses.

### Instructional design

In the 16-week experimental teaching, the data collection period was between September 2021 and December 2021. The explanation of the process, grouping method, and evaluation standards was done in the first week. The course schedule with thematic language teaching was followed from the second week. Finally, a general revision was conducted in the last week. Traditional teaching model is also preceded the course explanation, grouping method, and evaluation standard. The teaching starts from the second week as scheduled, and the general review is preceded in the last week.

In this study, a 16-week thematic teaching curriculum was implemented. The basic ideas of conceptual thematic teaching with the theory of English listening and speaking skills, student orientation with teacher support, emphasis on students’ learning background, and students’ interests are considered as the theme. In the research process, two thematic teaching activities were implemented, which are “animals,” with the theme of “animals” and “feelings,” with the theme of “emotion.” Two thematic teaching activities are preceded in the research process, including Animals, with “animal” as the theme, and “Feelings,” with “feeling” as the theme. The teaching experiment is preceded for 16 weeks; animals is preceded in the first 8 weeks, and feelings is preceded in the last 8 weeks. Each theme was implemented for 8 weeks, with a total of 24 sessions of instructional activity. Each main theme is accompanied with small topic discussions so that students can apply learning strategies for the acquisition of listening and speaking skills with teacher support.

Both thematic teaching and English learning theory emphasize authentic problems as learning content taking into account students’ interests and background knowledge. For this reason, Themes matching students’ learning interests and needs are selected for the self-designed materials, with the combination of rhythms and picture book stories with high repetition (How do you feel?). Songs from YouTube and self-made teaching texts were used in the implementation. The different learning styles which included group discussion, report, singing, and dancing were used to teach the two major themes. The “Animals” and “Feelings” themes were applied to improve students’ listening and speaking skills. In this study, the traditional teaching model was implemented by the traditional model of teachers who narrate textbooks.

The teaching process includes the following steps. (1) Revealing learning theme: Students are clearly informed the learning theme and course orientation to understand the course content and be attracted the interests as much as possible. The learning theme was determined based on the researcher’s investigation and observation in order to align the learning theme with the students’ interests. (2) Triggering motivation: Attracting students’ attention through pictures, stories, or songs to increase their motivation to learn. Through brainstorming and sharing between teachers and students, various theme-related learning materials were created organized, and classified to determine the sub-topics for discussions. (3) Getting into instructional activity: Multiple methods such as teacher lectures, class discussions, teamwork, hand painting, body movements, and presentation were used so that students can repeatedly practice listening and speaking skills while exploring the theme. (4) Evaluation and feedback: Teachers gave positive feedback on students’ presentations and integrated the thematic concept into the classroom.

Quantitative test is utilized for collecting data, which are objectively and justly interpreted and analyzed for the completion.

1. Worksheet: According to the demands for the course content, different worksheets are designed to check students’ listening and speaking learning conditions. The content combines thematic teaching and English listening and speaking evaluation.
2. Paper-and-pencil test and performance evaluation: In order not to have text reading become an obstacle for listening tests, the questions are presented with pictures to confirm pupils’ English listening learning performance. In terms of English speaking, the self-designed speaking evaluation questions are tested one-on-one between the teacher and the student. A self-designed speaking sheet for communication and interaction in English is also used for collecting students’ learning condition in speaking.

The measurement of listening comprehension and speaking skills in this study was conducted using worksheets, paper-and-pencil tests, and performance evaluation. Assessment refers to the teacher’s evaluation of the students, which is coded in statistics into very good (scored 81–100), good (scored 61–80), ordinary (scored 41–60), slightly low (scored 21–40), and extremely low (scored 0–20). These are shown in Likert scale as very good 5, good 4, ordinary 3, slightly low 2, and extremely low 1. Regarding learning anxiety, students filled out the learning anxiety scale after completing the course.

The learning anxiety scale in this study contains the following items. (1) I am not confident in speaking English in class. (2) I do not understand others’ (teachers or classmates) spoken English because I am nervous. (3) I am worried that I will not speak good English in class. (4) I am worried about giving wrong answers in English class. (5) I am afraid that I cannot understand what the teacher says in English class. (6) I feel shy and uncomfortable to speak English in front of my classmates. (7) I am afraid of speaking to the teacher in English. (8) I am worried about my weak English listening skills in tests. (9) I am worried that I cannot answer the questions in English tests because I read too slow. (10) I am nervous and frustrated because I do not understand the grammar in English tests. (11) I am worried about English tests even though I am well prepared. (12) I worried about questions that I cannot answer in English tests. (13) I am stressed in English tests because of the grammar rules. (14) I feel anxious when the English teacher corrects my English at any time. (15) I worried about failing English. (16) I am worried that people will laugh at my spoken English in English class. (17) I am ashamed of not speaking English fluently in class. (18) I am worried about being scolded by the teacher for my mistakes in practicing sentence patterns on stage. (19) I am worried about being scolded for poor performance on English tests.

### Data analysis method

Analysis of *t*-test was used to discuss the difference of thematic language teaching in listening and speaking skills and learning anxiety. Skaik (2015) explained that independent sample *t*-test was applicable when independent variables were two-way discrete variance and dependent variables were continuous variables. SPSS with *t*-test is therefore applied in this study to discuss the effect of phenomenon-based English teaching method on English listening, English speaking ability, and learning anxiety.

## Results

### Difference analysis of thematic language teaching and listening

According to the analysis of *t*-test, the effect of thematic language teaching on listening was discussed, i.e., analysis and explanation of teaching style. Thematic language teaching shows higher listening achievement than the traditional teaching model (Table 1). Therefore, it can be claimed that H1 was supported.

**Table 1:** Difference analysis of thematic language teaching in listening.

Variable		F	*P*	Analysis results
Thematic Language teaching	Listening	15.341	0.000^**^	Thematic Language teaching (3.89) > Traditional teaching model (3.43)

** *p* < 0.01.

### Difference analysis of thematic language teaching and speaking skill

The effect of thematic language teaching on speaking skill is discussed according to the analysis of *t*-test, i.e., analysis and explanation of teaching style. Table 2 shows that thematic language teaching (3.77) results in higher speaking skill achievement than the traditional teaching model (3.26). Accordingly, H2 was supported.

**Table 2:** Difference analysis of thematic language teaching and speaking skill.

variable		F	*P*	Analysis results
Thematic Language teaching	Speaking skill	18.556	0.000^**^	Thematic language teaching (3.77) > traditional teaching model (3.26)

** *p* < 0.01.

### Difference analysis of thematic language teaching and learning anxiety

The effect of thematic language teaching on learning anxiety was analyzed with an analysis of *t*-test. Table 3 shows lower learning anxiety after thematic language teaching (3.35) than before thematic language teaching (3.81). Therefore, H3 was supported.

**Table 3:** Difference analysis of thematic language teaching and learning anxiety.

variable		F	*P*	Analysis results
Thematic language teaching	Learning anxiety	28.441	0.000^**^	Traditional teaching model (3.81) > Thematic language teaching (3.35)

** *p* < 0.01.

## Discussion

In thematic language teaching, multimedia design students can explore different sub-topics under the theme (Shuqair and Dashti, 2019). Considering this, sub-topics in which students have an interest can be collected through worksheets or questionnaires, before designing curriculum. The curriculum planning, collaborative lesson preparation, and brainstorming with peers allow to include different learning activities such as group discussions, real-life operations, and challenging tasks, so that the target language can constantly be experienced in different authentic situations. Besides, collaborative development of assessment rules allows in-class learning activities to be a part of multiple assessments. In the practice of the thematic curriculum, teachers need to adjust and support teaching according to students’ learning situations to increase students’ exposure to language and improve their language proficiency (Lambić et al., 2021). Activities such as participation in activities at school such as reader’s theater, weekly sentence formation, songs, and word recitation can be considered as examples. Practicing through activities can increase learning opportunities and reduce language anxiety. Since thematic language teaching is an interdisciplinary teaching activity, teachers often need to collect data and look for teaching strategies to solve problems in the classroom (Cheung and Wang, 2019). In addition to reviewing teaching theories and looking for appropriate teaching strategies, they need to expand their knowledge and competencies in other fields to facilitate the teaching process. In this way, teachers can seek collaboration with colleagues to form a teacher community for communication, collaboration, and brainstorming (Strouse et al., 2018). The teachers collaboratively develop thematic curricula for thematic language teaching practice, as well as apply the curricula in other fields to maximize interest in the curriculum.

## Conclusion

The research findings revealed that multimedia design students showed development after the thematic language teaching in terms of speaking skill. Sari et al. (2019) mentioned that the use of thematic teaching can improve students’ speaking skill. They added that meaningful teaching activities played an important role in promoting students’ speaking skill. The use of thematic teaching can improve students’ oral performance, in the task completion process, and the intensive use of English can promote pupils’ speaking skills (Ahn et al., 2019). Thematic language teaching includes stories, songs, and sub-topic exploration. The learning activities include dynamic and static learning, such as group discussion, mood cards, dance, category posters, and role-play, to meet the demands of multimedia design students with different learning styles. Multimedia design students learn speaking in the target language through modeling. Their models are the teachers, characters in the videos, or their peers. Various learning activities provide natural, rich, and diverse authentic vocabulary learning situations for multimedia design students’ constant use of language. By so doing, they move from the mechanical speaking practice of repeating after their teachers and language learning then becomes meaningful for daily life. With thematic language teaching, multimedia design students make progress in learning listening skill. Wardani et al. (2020a) found that thematic teaching can promote students’ listening comprehension and learning attitude. Students who were trained through English as a medium of instruction (EMI) presented better English listening comprehension than those who were trained in L1. Thematic teaching revealed a positive effect on listening comprehension attitude and willingness to give positive feedback (Normand and Burji, 2020). The research results, in this study, are similar to what Wardani et al. (2020b) found in their study. They stated that thematic teaching can promote the effectiveness of the course. Employing a variety of teaching styles and methods can enrich students’ learning experience. Students with distinctive learning achievement levels can be promoted remarkably through effective learning in thematic teaching which includes game elements. Immersion in context can facilitate learners’ practice and comprehension of the target language vocabulary. The selection of learning content for the listening skill is mainly based on students’ interests or life experiences. In this way, their listening comprehension skill was improved as well as their learning motivation and interests were positively affected. These, in turn, greatly reduced the learning anxiety (Normand and Burji, 2020). In thematic language teaching, teachers select stories that use sentences with high repetition, body movements, and role-play, to help multimedia design students’ listening comprehension. Designing listening tasks with clear steps, such as taking pictures while listening to vocabulary and drawing emotional expressions while listening to sentences, can improve multimedia design students’ listening comprehension. It is difficult to target multimedia design students’ listening comprehension because learners’ spoken and non-spoken responses provide important information for teachers’ evaluation of multimedia design students’ comprehension.

## Data availability statement

The original contributions presented in the study are included in the article/supplementary material, further inquiries can be directed to the corresponding author.

## Ethics statement

This study was reviewed and approved by the Ethics Committee of the Cheng Shiu University. Written informed consent was obtained from all participants for their participation in this study.

## Author contributions

S-KY performed the data collection and data analysis and approved the submitted version of the manuscript.

## Conflict of interest

The author declares that the research was conducted in the absence of any commercial or financial relationships that could be construed as a potential conflict of interest.

## Publisher’s note

All claims expressed in this article are solely those of the authors and do not necessarily represent those of their affiliated organizations, or those of the publisher, the editors and the reviewers. Any product that may be evaluated in this article, or claim that may be made by its manufacturer, is not guaranteed or endorsed by the publisher.

## References

[ref1] Ahn S. J. Johnsen K. Ball C. (2019). Points-based reward Systems in Gamification Impact Children’s physical activity strategies and psychological needs. Health Educ. Behav. 46, 417–425. doi: 10.1177/1090198118818241, PMID: 30678507 PMC6566098

[ref2] Bezerra Fagundes T. (2016). Concepts of the teacher as researcher and reflective teacher: perspectives about teachers’ work. Rev. Bras. Educ. 21, 281–298. doi: 10.1590/S1413-24782016216516

[ref3] Blazar D. (2015). Effective teaching in elementary mathematics: identifying classroom practices that support student achievement. Econ. Educ. Rev. 48, 16–29. doi: 10.1016/j.econedurev.2015.05.005

[ref4] Chang C. Lin H. C. K. (2019). Classroom interaction and learning anxiety in the IRs-integrated flipped language classrooms. Asia Pac. Educ. Rev. 28, 193–201. doi: 10.1007/s40299-018-0426-x

[ref5] Chen Y. Chang C. C. (2018). The impact of an integrated robotics STEM course with a sailboat topic on high school students’ perceptions of integrative STEM, interest, and career orientation. Eurasia J. Math. Sci. Technol. Educ. 14:1614. doi: 10.29333/ejmste/94314

[ref6] Cheung S. K. S. Wang F. L. (2019). Blended learning in practice: guest editorial. J. Comput. High. Educ. 31, 229–232. doi: 10.1007/s12528-019-09229-8

[ref7] Chumdari S. A. Budiyono N. S. (2018). Implementation of thematic instructional model in elementary school. Int. J. Educ. Res. Rev. 3, 23–31. doi: 10.24331/ijere.413964

[ref8] Haryanto A. Sukardi W. Nashruddin N. (2018). Politeness principle and its implication in EFL classroom in Indonesia. XLinguae 11, 99–112. doi: 10.18355/XL.2018.11.04.09

[ref9] Hennessey B. Hooper L. E. Leavitt C. L. (2018). Realisms and idealisms in Italian culture, 1300-2017. Italianist 37, 281–288. doi: 10.1080/02614340.2017.1409308

[ref10] Hsu T. C. (2019). Using a concept mapping strategy to improve the motivation of EFL students in Google hangouts peer-tutoring sessions with native speakers. Interact. Learn. Environ. 27, 272–285. doi: 10.1080/10494820.2018.1463268

[ref001] Horwitz E. K. Horwitz M. B. Cope J. (1986). Foreign language classroom anxiety. Mod. Lang. J. 70, 125–132. doi: 10.1111/j.1540-4781.1986.tb05256.x

[ref11] Huang S. (2018). Authentic texts as reading materials in a Chinese as a foreign language classroom: learners’ perceptions and pedagogical implications. J Natl Counc Less Common Taught Lang 23, 1–40.

[ref12] Lambić D. Đorić B. Ivakić S. (2021). Investigating the effect of the use of code. Org on younger elementary school students’ attitudes towards programming. Behav. Inf. Technol. 40, 1784–1795. doi: 10.1080/0144929X.2020.1781931

[ref13] Lan Y. J. Liao C. Y. (2018). The effects of 3D immersion on CSL students’ listening comprehension. Innov. Lang. Learn. Teach. 12, 35–46. doi: 10.1080/17501229.2018.1418242

[ref14] Lin Y. H. Tseng Y. C. (2020). Learning English in tourism and hospitality internships overseas: reflections from six Taiwanese college students. Int J Eng. Linguistics 10:1. doi: 10.5539/ijel.v10n5p1

[ref15] Maulana A. Musthafa I. Hayati T. N. (2020). The efficiency of teaching listening and speaking skills to develop Students' communicative competences. Univ. J. Educ. Res. 8, 802–808. doi: 10.13189/ujer.2020.080310

[ref16] Moreno Martínez N. M. López Meneses E. Leiva Olivencia J. J. (2018). El uso de las tecnologías emergentes como recursos didácticos en ámbitos educativos. Int. Stud. Law Educ. 29, 131–146.

[ref17] Normand M. P. Burji C. (2020). Using the step it UP! Game to increase physical activity during physical-education classes. J. Appl. Behav. Anal. 53, 1071–1079. doi: 10.1002/jaba.624, PMID: 31414481

[ref18] Oxford R. (1990). Language learning strategies: What every teacher should know. New York: Newbury House Publishers.

[ref19] Romanova A. (2017). Listening comprehension: facilitating strategies. Euromentor J. 8, 65–75.

[ref20] Sälzer C. Roczen N. (2018). Assessing global competence in PISA 2018: challenges and approaches to capturing a complex construct. Int. J. Dev. Educ. Global Learn. 10, 5–20. doi: 10.18546/IJDEGL.10.1.02

[ref21] Sari F. K. Rakimahwati R. Fitria Y. (2019). Development of 2013 curriculum integrated thematic teaching materials with a scientific approach in class 1 elementary school. Int. J. Educ. Dyn. 1, 125–131. doi: 10.24036/ijeds.v1i2.128

[ref22] Shuqair K. Dashti A. (2019). Teachers' perceptions of the use and effectiveness of Children's literature in the EFL classrooms of the primary schools of Kuwait. Engl. Lang. Teach. 12, 87–97. doi: 10.5539/elt.v12n7p87

[ref23] Skaik Y. (2015). The bread and butter of statistical analysis "t-test": uses and misuses. Park. J. Med. Sci. 31, 1558–1559. doi: 10.12669/pjms.316.8984, PMID: 26870136 PMC4744321

[ref24] Solgi F. Tafazoli D. (2018). The necessity of teaching culture in English as a foreign language course: Iranian perspective. J. Lang. Linguist. Stud. 14, 1–11.

[ref25] Strouse G. A. Nyhout A. Ganea P. A. (2018). The role of book features in young children's transfer of information from picture books to real-world contexts. Front. Psychol. 9:50. doi: 10.3389/fpsyg.2018.00050, PMID: 29467690 PMC5807901

[ref26] Wardani N. F. K. Sunardi Suharno (2020a). Thematic learning in elementary school: problems and possibilities. Adv. Soc. Sci. Educ. Humanit. Res. 397, 791–800. doi: 10.2991/assehr.k.200129.099

[ref27] Wardani N. F. K. Sunardi Suharno (2020b). Context-based thematic teaching materials to improve elementary students’ learning achievements. J. Pendidik. IPA Indones. 9, 193–202. doi: 10.23887/jpi-undiksha.v9i2.22822

[ref28] Wu T. T. (2018). Improving the effectiveness of English vocabulary review by integrating ARCS with mobile game-based learning. J. Comput. Assist. Learn. 34, 315–323. doi: 10.1111/jcal.12244

[ref29] Yeh Y. L. Lan Y. J. (2018). Fostering student autonomy in English learning through creations in a 3D virtual world. Educ. Technol. Res. Dev. 66, 693–708. doi: 10.1007/s11423-017-9566-6

